# Multi-resistant pseudomonas aeruginosa: study of risk factors for its detection at intensive care unit admission

**DOI:** 10.1186/2197-425X-3-S1-A123

**Published:** 2015-10-01

**Authors:** S Puerto Corrales, R Vara Arlanzón, F Callejo-Torre, JM Eiros Bouza, MJ Coma del Corral, ME Perea Rodríguez, M Martínez Barrios, M Gero Escapa, S Ossa Echeverri, M del Valle Ortíz, S Calvo Simal, E Portugal Rodríguez, JA Fernández Ratero, ENVIN-HELICS Study Group

**Affiliations:** Intensive Care, Hospital Universitario de Burgos, Burgos, Spain; Microbiology, Hospital Clinico Universitario de Valladolid, Valladolid, Spain; Hospital Universitario de Burgos, Burgos, Spain

## Introduction

For both infection control (avoiding dispersion of multi-resistant) and to ensure empirical antibiotic treatment is essential to be able to detect colonization/ infection by multi-resistant *Pseudomonas aeruginosa* (C/I- MRPa) at ICU admission.

## Objectives

Identify risk factors for easy detection (without requiring access to medical history) for C/I- MRPa at ICU admission. Evaluate whether with the factors chosen after literature review and analyzed, it is possible to develop an effective predictive model.

## Methods

From variables collected prospectively in ENVIN study (national nosocomial infection surveillance study), data was analyzed from 16950 patients admitted in 151 polyvalent ICU during the period from April to June in 2010, as a multipurpose cohort with univariable analysis and a multivariable logistic regression. Multi-resistant *Pseudomonas aeruginosa*, was defined as being resistant to three or more antipseudomonal families. The study variables are: age, gender, underlying disease, origin, urgent surgery, immunosuppression, immunodeficiency, neutropenia, surgical wound infection (superficial or deep) and skin and soft tissue infections.

## Results

In the univariable analysis we found statistical relationship between C/I-MRPa at admission time in ICU and the variables: underlying disease, origin, immunodeficiency, neutropenia, superficial surgical wound infection or skin and soft tissue infection. In the multivariate analysis we identified as independent risk factors: underlying medical disease (2,07(1,17-3,66) 0.01), immunosuppression (3,22(1,79-5,79) < 0.001) and superficial surgical wound infection (6,65(1,70-26) 0.006). Coronary artery disease acts as a protective factor (0.26 (0,07-0,92) 0.03). The predictive model globally considered, shows a moderate discriminating capacity (ABC-COR: 0.72;) 95% CI (0,67-0,78).

## Conclusions

There is an increase in the risk for C/I- MRPa at ICU admission in medical patients (2 times), immunosuppressed (3 times) or in those that present an infection of superficial surgical wound at the admission time in the unit (6 times). Coronary patients have lower risk. Despite assessing all variables described, we couldn´t develop a satisfactory predictive model because of its fair global prediction capability. Risk factors described should be taken into account for future studies with similar approach.

